# Validity and Test–Retest Reliability of Spatiotemporal Running Parameter Measurement Using Embedded Inertial Measurement Unit Insoles

**DOI:** 10.3390/s24165435

**Published:** 2024-08-22

**Authors:** Louis Riglet, Baptiste Orliac, Corentin Delphin, Audrey Leonard, Nicolas Eby, Paul Ornetti, Davy Laroche, Mathieu Gueugnon

**Affiliations:** 1 CHU Dijon–Bourgogne, Centre d’Investigation Clinique, Module Plurithématique, Plateforme d’Investigation Technologique, 21000 Dijon, France; louis.riglet@chu-dijon.fr (L.R.); paul.ornetti@chu-dijon.fr (P.O.); davy.laroche@u-bourgogne.fr (D.L.); 2 INSERM, CIC 1432, Module Plurithématique, Plateforme d’Investigation Technologique, 21000 Dijon, France; 3 Zhortech SAS, 54000 Nancy, France; 4 INSERM, UMR1093-CAPS, Université Bourgogne Franche-Comté, UB, 21000 Dijon, France; 5 Rheumatology Department, CHU Dijon–Bourgogne, 21000 Dijon, France; 6 Collaborative Research Network STARTER (Innovative Strategies and Artificial Intelligence for Motor Function Rehabilitation and Autonomy Preservation), 21000 Dijon, France

**Keywords:** running gait analysis, insole, validation, repeatability, 3D motion analysis, running

## Abstract

Running is the basis of many sports and has highly beneficial effects on health. To increase the understanding of running, DSPro^®^ insoles were developed to collect running parameters during tasks. However, no validation has been carried out for running gait analysis. The aims of this study were to assess the test–retest reliability and criterion validity of running gait parameters from DSPro^®^ insoles compared to a motion-capture system. Equipped with DSPro^®^ insoles, a running gait analysis was performed on 30 healthy participants during overground and treadmill running using a motion-capture system. Using an intraclass correlation coefficient (ICC), the criterion validity and test–retest reliability of spatiotemporal parameters were calculated. The test–retest reliability shows moderate to excellent ICC values (ICC > 0.50) except for propulsion time during overground running at a fast speed with the motion-capture system. The criterion validity highlights a validation of running parameters regardless of speeds (ICC > 0.70). This present study validates the good criterion validity and test–retest reliability of DSPro^®^ insoles for measuring spatiotemporal running gait parameters. Without the constraints of a 3D motion-capture system, such insoles seem to be helpful and relevant for improving the care management of active patients or following running performance in sports contexts.

## 1. Introduction

Running is among the most popular physical activities, and it is the basis of many sports or leisure activities. The practice of running, outside or on a treadmill, has greatly increased in recent years [1] with high health benefits [2,3]. To evaluate its effects and improve performance [4,5], analyzing running patterns is increasingly performed. Traditionally, rating scales and subjective observations are used for this analysis, but these approaches are less sensitive to performance changes in training or injury [6,7].

To provide objective, reliable, and reproducible outcomes, running gait analysis can be performed using 3D motion capture [8]. This non-invasive motion system is considered to be a gold standard for walking and running gait analysis [9,10,11] and helps to measure locomotion in depth through spatiotemporal, kinematic, and kinetic parameters. However, the principal disadvantages of this system are its long operation time, dedicated space requirements, technical expertise requirements, and high costs, preventing an assessment of running in an ecological context [9]. Significant progress has been made with the development of markerless systems and smartphone apps for performing gait analysis in clinical and sports contexts [12,13,14]. They have the advantage of reducing data collection and processing time [13]. However, these systems have limitations, including limited capture volume (based on the number of cameras) or partial accuracy (e.g., joint angle estimation) [12,13].

To overcome these 3D gait recording limitations, wearable devices are increasingly accepted and used by runners and clinicians [15,16,17]. Wearable systems (Inertial Measurement Unit—IMU) are based on a combination of accelerometers, gyroscopes, and magnetometers, allowing the description of normal or pathological human locomotion in a wide variety of environments [18]. In running gait analysis, IMUs help to measure running gait parameters, notably spatiotemporal parameters, and quantify running performance [19,20]. Despite their good feasibility and performance, there are major remaining technical disadvantages, such as sensor attachment errors, external signal noise, signal filtering errors, and integration drift [18].

To record foot movements and orientations, IMUs can be fixed on or under the foot. In particular, DSPro^®^ insoles, developed by DigitSole SAS, have the advantage of being able to collect data during different tasks. For instance, this device has been validated for walking gait analysis compared to 3D motion analysis [21]. Recent studies of this device have highlighted good repeatability in the measurement of walking gait parameters such as cadence, walking speed, and stride length [21,22,23]. However, the validation of the extracted parameters during running is a prerequisite for extending their use in this activity.

For this reason, the aim of this present study was to evaluate the test–retest reliability and criterion validity (i.e., the extent to which scores for volunteers who have not changed are the same for repeated measurements over time and the degree to which the scores of Patient-Reported Outcome Measures (PROMs) are an adequate reflection of a “gold standard”, respectively [24]), of the running parameters measured using embedded insoles in comparison to those obtained from the gold-standard system, i.e., a motion analysis system.

## 2. Materials and Methods

### 2.1. Population

A monocentric study was performed from October 2021 to February 2022 and included 30 healthy participants [21] who provided informed oral consent. The local institutional ethics committee and the French National Agency for Drug Safety approved and authorized the study protocol. The Clinical Trial registration reference is NCT05104645.

### 2.2. Procedure and Materials

**Figure 1:**
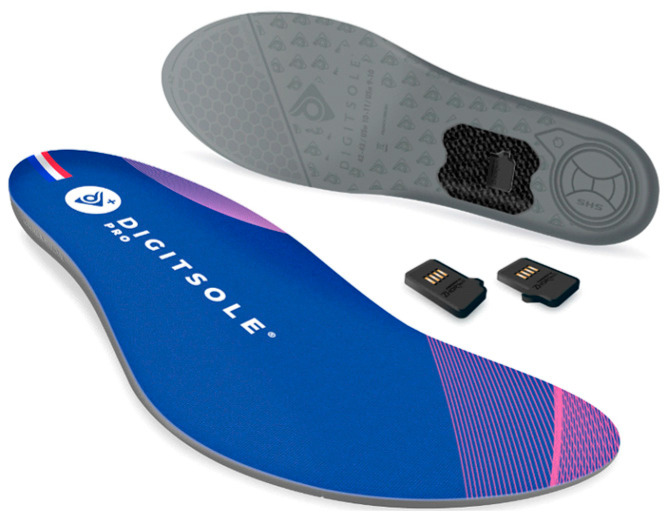
DSPro^®^ insole device.

The procedure and materials used in this study were described by Riglet et al. [21]. Briefly, the experiments were carried out at the INSERM U1093 laboratory (Dijon University, France). Each participant participated in two visits, with the second visit occurring seven days after the first. Motion analysis was carried out using Nexus software (Vicon System^®^, Oxford, UK, 2.12.1 version), and the volunteers were equipped with the Conventional Gait Model (version 2.5) markers set [25,26]. One marker was added at the extremity of the shoe, near the hallux. The 3D position of each marker was tracked with 18 optoelectronic cameras (11 VERO and 7 MX-T10 cameras, Vicon System^®^, Oxford, UK; 100 Hz). Two force plates (AMTI^®^, Watertown, MA, USA; 1000 Hz) were embedded in the floor to record ground reaction forces. The volume of interest was approximately 10 m × 2 m × 2.5 m, with an error threshold set to 0.5 mm in accordance with manufacturer data. Additionally, each participant wore identical shoes (Ekiden One, Kalenji^®^, Villeneuve d’Ascq, France) fitted with DSPro^®^ insoles (Zhortech^®^ algorithms, DigitSole SAS, Nancy, France, 104 Hz) (Figure 1). A sensor integrating accelerometers, gyroscopes and magnetometers was fixed onto the proximal part of each insole, allowing the calculation of linear accelerations and angular velocities in three dimensions.

Participation involved two running conditions (overground and treadmill running) at two different speeds. Participants were asked to run on a 10-m walkway at a self-selected speed (comfortable) and then at a fast speed. These running overground tasks were performed at least five back-and-forth runs for each speed. In the second condition, participants were asked to run on a treadmill at a self-selected speed (comfortable speed obtained after a 2-min familiarization trial), then at a fast speed (adding 2 km/h to the comfortable speed).

### 2.3. Data Analysis

The synchronization between the two systems (IMUs and motion capture) was synchronized using an analog button to trigger the beginning of each running trial. For each participant, a minimum of 5 trials for running overground and 1 trial of 2 min for treadmill running were collected (for comfortable and fast speed).

For motion capture, lower limb markers and ground reaction forces were post-processed following the method described in Riglet et al. [21]. Briefly, gait events (heel strike and toe off) were computed using the position of the foot velocity algorithm by O’Connor et al. [25]. The maximum vertical foot velocity corresponds to the toe off and the minimum to heel strike. Then, two other gait parameters were computed: flat foot in (FFI) and flat foot out (FFO), corresponding, respectively, to the sample in which the toe touches the ground after the heel strike and to the sample in which the heel takes off the ground after heel strike (please see Riglet et al. [21] for more details). For insoles, these events were also extracted (Zhortech properties). A comparison of the initial heel strike of each gait cycle between motion capture and insoles was performed and considered to be synchronized if the difference was less than 0.35 s. Custom-made scripts with Python (3.9) and Matlab (MathWorks^®^, Natick, MA, USA, R2023a) were used to perform all post-processing.

Regardless of running conditions, the parameters of interest were speed, stride cadence, flight time, stance time, stride time, swing time, stride length, stride height, and plantar flexion angle foot in. For overground running, loading time, propulsion time, impact force, and leg stiffness were also measured based on force plate data. Without an instrumented treadmill, these parameters were not measured for treadmill conditions. The definition of running parameters measured with motion capture is detailed in Table S1 (definition of parameters measured with insoles are not available: property of Zhortech company).

### 2.4. Statistical Analysis

Based on the comparison of each running parameter measured during two sessions, test–retest reliability was estimated. As suggested by Koo & Li [26], estimation was performed using intraclass correlation (ICC) and its 95% confidence interval (CI) based on a mean-rating, absolute agreement, 2-way mixed-effects model. For motion capture and insoles, ICC and *p*-value (*p*) were computed between the two sessions for each running condition. Additionally, absolute and relative errors between sessions were computed, and a comparison between motion capture and insoles was performed using a Student’s t-test. Based on the standard error of measurement (SEM) and the standard deviation (SD), minimal detectable change (MDC) was also computed for each variable and condition [27] and was defined as follows:

$$MDC=1.96.SEM.\sqrt{2}\;\mathrm{with}\;SEM=SD\sqrt{1-ICC}.$$

Based on the comparison of each running parameter measured by motion capture and insoles, criterion validity was estimated. As suggested by Koo & Li [26], estimation was performed using intraclass correlation based on a mean-rating, consistency, 2-way mixed-effects model (regardless of the session and gait side (right and left) for both systems). Additionally, a Lin concordance coefficient (CCC) and Bland–Altman analysis were also carried out. Based on Bland–Altman plots, limits of agreement (LoA) were calculated and defined as the mean difference between two measurements ± 1.96 SD of the difference.

ICC values were classified into four categories: poor (lower than 0.5), moderate (between 0.5 and 0.75), good (between 0.75 and 0.9), and excellent (higher than 0.9) [26]. Using the Bonferroni correction, the threshold of significance was fixed at *p* < 0.004 (0.05 divided by the number of variables (13)). CCC values were considered to be excellent when the coefficient was higher than 0.8 [28]. All statistical analyses were performed using Matlab (MathWorks^®^, R2023a).

## 3. Results

### 3.1. Population Characteristics

The population was composed of 14 females and 16 males. Female population characteristics were age 27.6 ± 5.2 years, height 165.2 ± 5.3 cm, body mass 61.0 ± 9.2 kg and shoe size 39.2 ± 1.8. Male population characteristics were age 28.2 ± 6.1 years, height 180.3 ± 5.3 cm, body mass 74.4 ± 8.6 kg and shoe size 43.4 ± 1.5.

### 3.2. Running Gait Cycle

For overground running, 2896 gait cycles were recorded (comfortable speed: 1640 cycles and fast speed: 1256 cycles). For treadmill running, 18,945 gait cycles were recorded (comfortable: 9317 cycles and fast: 9628 cycles).

### 3.3. Test–Retest Reliability

For overground running, the mean and standard deviation of each parameter, ICC values, and MDC are presented in Table 1 for motion capture and Table 2 for insoles.

For motion capture, ICC values were considered excellent for swing time, impact force, and leg stiffness at a comfortable speed and for flight time, stride length, and impact force at a fast speed. Speed, stance time, propulsion time, stride length, and stride height at comfortable speed had a moderate ICC value. The ICC values for the other parameters were considered to be good. Concerning fast speed, the ICC values for speed, swing time, and stride height were good. A lower ICC value was found for propulsion time (ICC = 0.499, *p* = 0.004). The other parameters had moderate ICC values.

For insole devices, ICC values were considered excellent for impact force and leg stiffness at a comfortable speed and for flight time, impact force, and leg stiffness at a fast speed. For comfortable speed, the following parameters had ICC values higher than 0.75: flight time, stance time, swing time, and flexion angle foot in. Fast speed, swing time, stride length, and stride height also had ICC values higher than 0.75. Regardless of speed conditions, the other parameters had moderate ICC values.

Concerning treadmill running, the mean and standard deviation of each parameter, ICC values, and MDC are presented in Table 3 for motion capture and Table 4 for insoles. For motion capture, parameters were significantly correlated between sessions with good to excellent ICC values (0.853 to 0.999, *p* < 0.001). For insole devices, parameters were significantly correlated between sessions with good to excellent ICC values (0.875 to 0.995, *p* < 0.001) regardless of speed.

The absolute and relative error between the two sessions of each parameter extracted with motion capture and with insoles are presented in Tables S2 and S3. No significant difference was found, except for stride height during treadmill running (fast condition, *p* = 0.002).

### 3.4. Criterion Validity

**Figure 2:**
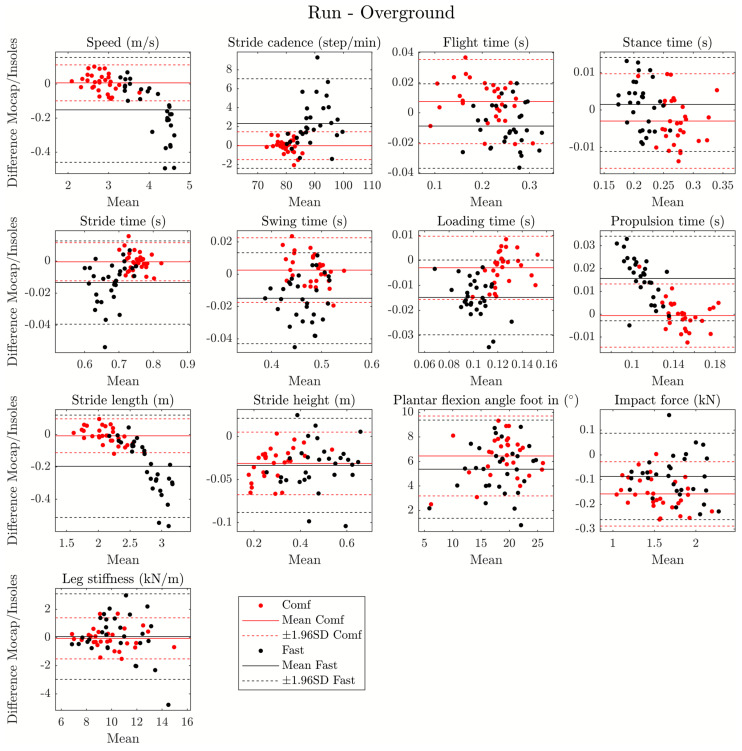
Bland–Altman plot for all running parameters during fast (black points) and comfortable (red points) overground running. Solid line = mean, dashed line = ±1.96 SD.

**Figure 3:**
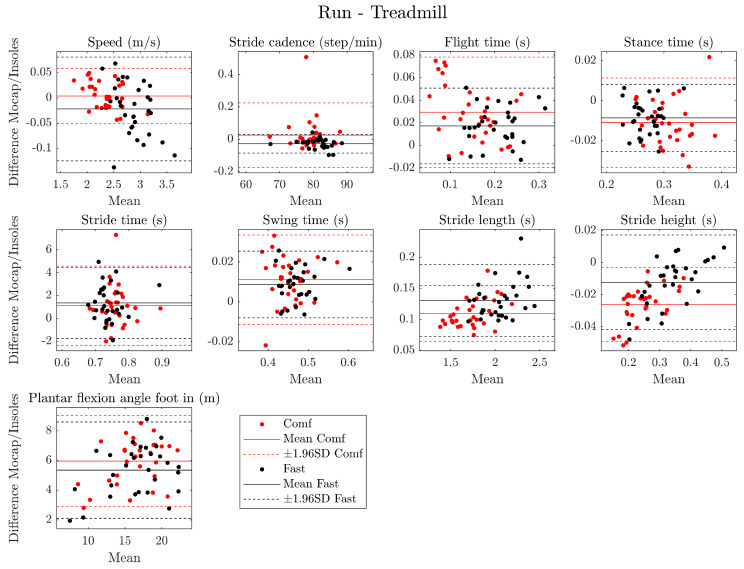
Bland–Altman plot for all running parameters during fast (black points) and comfortable (red points) treadmill running. Solid line = mean, dashed line = ±1.96 SD.

Mean, standard deviation, and ICC values for running parameters are presented in Table 5 for the overground and Table 6 for the treadmill. Additionally, Bland–Altman plots for running parameters are presented in Figure 2 for the overground and Figure 3 for the treadmill. CCC values and plots are presented in Table S4 and Figures S1 and S2.

Concerning intraclass correlation, ICC values from 0.729 to 0.987 (*p* < 0.001) were found for all parameters in overground running (comfortable and fast speed). For fast speed, leg stiffness had a moderate intraclass correlation (ICC < 0.75, *p* < 0.001). The other parameters showed good and excellent intraclass correlations (ICC > 0.75, *p* < 0.001). For treadmill running, ICC values from 0.899 to 0.999 (*p* < 0.001) were found for all parameters (comfortable and fast speed).

Concerning Lin concordance coefficient, CCC values were considered excellent (higher than 0.8) for all parameters except for plantar flexion foot during all speeds in overground and during treadmill running (CCC = [0.358–0.500]) as well as loading time (CCC = 0.467), propulsion time (CCC = 0.453) and stride length (CCC = 0.690) for fast speed during overground running.

Based on Bland–Altman plots, the following temporal parameters were found to have a mean bias close to zero seconds: flight time, stride time, stance time, swing time, loading time, and propulsion time. The following spatial parameters also have a mean bias close to zero: speed, stride cadence, stride length, and stride height. These spatial parameters also showed an increase in error heterogeneity with speed. The mean bias for plantar flexion angle foot was close to 6°. During overground running, the mean bias for dynamic parameters (impact force and leg stiffness) was close to −0.1 kN and 0 kN/m. Moreover, LoA was found to be lower than 15% of mean values for all parameters except plantar flexion foot in (20.5% and 25.2%, respectively, for comfortable and fast speed) and leg stiffness (27.9% for fast speed) for overground running. For treadmill running, flight time had an LoA of 26.6% for comfortable speed and 18.2% for fast speed. Plantar flexion foot in had an LoA of 20.6% for comfortable speed and 21.8% for fast speed.

## 4. Discussion

The aim of the present study was to evaluate the test–retest reliability and criterion validity of running parameters measured using embedded insoles in comparison to the values obtained from the gold standard motion-capture system. Based on the motion capture of 30 participants during overground and treadmill running, the present results highlight the relevance of DSPro^®^ insoles in a sports context. Furthermore, the evaluation of two distinct speeds for both overground and treadmill running introduced a novel dataset, enhancing the precision and comprehensiveness of the validation process for these insoles.

Test–retest reliability was measured to quantify the degree of agreement of parameters for treadmill and overground running. For overground running, ICC values varied between moderate and excellent reliability. The moderate ICC values were explained by the high variability in running between two specific sessions overground because, unlike treadmill running, participants could choose their speed during the second session, thus modifying the gait parameters. However, the results obtained for insoles and motion capture on the treadmill highlight good to excellent reliability due to the identical speed setting between the two sessions. Nevertheless, propulsion time for motion capture shows a moderate and poor ICC value for comfortable and fast speed, respectively. Only a few previous studies have examined the reliability of measurements derived from IMUs during running [29], but our results are comparable to previous research, regardless of the conditions. For example, Deflandre et al. [30] reported good to excellent reliability for stance time and step frequency with a portable accelerometer during treadmill running. Using textile socks incorporating IMU, Mason et al. [31] also highlighted good to excellent test–retest agreement for stride and swing time during treadmill running and moderate to good during overground running. Additionally, the good results of this present study were reinforced by the absence of significant differences in absolute and relative errors between the two sessions (except for stride height during treadmill running in the fast condition), and all these values were lower than the MDC.

For criterion validity, good results were obtained with the ICC analysis. Spatiotemporal parameter measurements had good to excellent agreement between insoles and the motion-capture system regardless of speed (except for leg stiffness at fast speed). Additionally, these results were observed for treadmill conditions with ICC values higher than 0.80 for all parameters. The good reliability of DSPro^®^ is consistent with other validation studies of IMU against a 3D motion analysis system during a running task. For instance, Uno et al. [32] found excellent relative validity (ICC > 0.900) for stride length, stride duration, stride frequency, stride speed, vertical height, stance phase duration, swing phase duration, and sagittal angle. Brahms et al. [33] found an ICC value of 0.955 for stride length during running. Moreover, in a recent meta-analysis, Horsley et al. [34] reported similar ICC values for stance phase time (ICC = 0.81–0.97), swing phase time (ICC = 0.56–0.81), stride duration (ICC = 0.55–0.99), stride frequency (ICC = 0.96–0.99), stride length (ICC = 0.75–0.99), and flight time (ICC = 0.81–0.86). Additionally, CCC values were in accordance with ICC values except for loading time (fast speed—overground running), propulsion time (fast speed—overground running), and plantar flexion foot in (comfortable and fast speed—overground and treadmill running). Differences between these two coefficients could be linked to deviations of the point cloud from the identity line. Finally, the Bland–Altman plots show that there is low heterogeneity in the measurement bias, with a mean bias close to 0 for spatiotemporal parameters, impact force, and leg stiffness in overground and treadmill conditions. Moreover, a bias of 6° was observed for the plantar flexion angle foot in but not influenced by the speed of running, and a bias of about 20 cm was observed for stride length during fast speed. Additionally, a linear error was observed for speed, stride cadence, stride time, propulsion time, and stride length during overground running. All these biases/errors could be partially explained by the hardware calibration and the algorithm used (fusion filters and calculation method) between motion capture and embedded IMU insoles. Additionally, LoA were not negligible but seem satisfactory with values lower than 15% compared to the mean. However, due to LoA higher than 20%, plantar flexion foot in was only partially validated for all conditions, flight time for treadmill condition, and leg stiffness for overground running at fast speed. However, these variables had moderate to excellent ICC values.

Furthermore, this study validates the use of IMU embedded insoles during overground and treadmill running and, since running was performed in different conditions and at different speeds, indicates that it is a clinically relevant means of assessing gait performance. IMUs are low-cost systems and could easily be used to follow different running parameters for professionals or individuals. Additionally, considering the limitations of motion capture (dedicated space requirements, technical expertise requirements, and high costs) [9], running analysis could be easily performed only with insoles in a clinical context.

This study has some limitations. First, running experiments were conducted in a laboratory, which can modify kinematics and temporal variables compared to outdoor running. Many studies have highlighted the importance of measuring running outside of the laboratory in natural training and/or competition environments [35]. Moreover, the treadmill is the most common system used to evaluate and quantify running gait [17]. This approach has the advantage of providing a standardized and reproducible environment. However, treadmill running only partially reflects natural running behavior, and there are differences in kinematic and kinetic patterns compared to overground running [15,17,36]. Additionally, without an instrumented treadmill and due to the lack of international consensus definition, the loading time parameter was not measured for this condition. However, depending on the attack of the foot during treadmill running, there is a potential for considerable variability, and comparison with data from insoles seems difficult. Additionally, a choice has been made not to estimate leg stiffness and impact force with literature models (e.g., presented by Morin et al. [37]) in order to validate insoles with real measurements. Second, experiments involved young (mean 27.6 ± 5.2 years) and healthy participants. Showing good accuracy for this population, these results could be modified for older or injured populations. Further investigations are warranted in patients with locomotor disabilities (e.g., with a higher risk of falls) to confirm these good psychometric properties of the insoles in care settings (neurologic rehabilitation, orthopedic surgery, etc.).

## 5. Conclusions

To conclude, by comparing running parameters measured with insoles and a motion-capture system, this present study confirms the good accuracy and repeatability of DSPro^®^ insoles for most running parameters. Although a few parameters, such as plantar flexion foot in, flight time, and leg stiffness, were only partially validated under specific conditions, this wearable device seems to be a useful tool to improve the understanding of running performance. These insoles are a relevant way to overcome the constraints of the motion-capture system, and they have the potential to improve patient care and enhance the analysis of running performance in a sports context.

## Figures and Tables

**Table 1:** Mean, standard deviation, ICC values (with 95% CI), and minimal detectable change (MDC) of running parameters were measured using a motion-capture system for overground conditions. Significant results are indicated as follows: * = *p* < 0.001, ^+^ = *p* < 0.004.

	OVERGROUND RUNNING—MOTION CAPTURE							
	Comfortable Speed				Fast Speed			
	Mean ± SD		ICC [95% CI]	MDC	Mean ± SD		ICC [95% CI]	MDC
	Session 1	Session 2			Session 1	Session 2		
Speed (m/s)	2.75 ± 0.44	2.87 ± 0.34	0.549 [0.238; 0.761] ^+^	0.73	4.14 ± 0.61	4.17 ± 0.55	0.776 [0.581; 0.887] *	0.75
Stride Cadence (step/min)	79.89 ± 3.12	80.16 ± 3.07	0.850 [0.705; 0.928] *	3.29	87.81 ± 6.10	87.87 ± 5.19	0.720 [0.489; 0.857] *	8.23
Flight Time (s)	0.20 ± 0.06	0.22 ± 0.06	0.837 [0.637; 0.926] *	0.07	0.26 ± 0.04	0.26 ± 0.04	0.912 [0.824; 0.957] *	0.04
Stance Time (s)	0.28 ± 0.03	0.27 ± 0.03	0.723 [0.454; 0.866] *	0.04	0.21 ± 0.02	0.21 ± 0.02	0.746 [0.531; 0.871] *	0.03
Stride Time (s)	0.75 ± 0.03	0.75 ± 0.03	0.847 [0.699; 0.926] *	0.03	0.68 ± 0.05	0.68 ± 0.04	0.704 [0.463; 0.848] *	0.07
Swing Time (s)	0.48 ± 0.04	0.48 ± 0.04	0.903 [0.784; 0.956] *	0.03	0.47 ± 0.04	0.47 ± 0.04	0.839 [0.688; 0.920] *	0.04
Loading Time (s)	0.13 ± 0.01	0.13 ± 0.01	0.757 [0.539; 0.881] *	0.02	0.11 ± 0.01	0.11 ± 0.02	0.620 [0.316; 0.809] *	0.02
Propulsion Time (s)	0.15 ± 0.02	0.14 ± 0.02	0.524 [0.164; 0.756] ^+^	0.04	0.11 ± 0.02	0.10 ± 0.02	0.499 [0.152; 0.738]	0.04
Stride Length (m)	2.08 ± 0.33	2.17 ± 0.29	0.657 [0.385; 0.825] *	0.51	2.85 ± 0.38	2.87 ± 0.35	0.904 [0.809; 0.953] *	0.31
Stride Height (m)	0.30 ± 0.09	0.33 ± 0.08	0.725 [0.473; 0.865] *	0.12	0.49 ± 0.11	0.50 ± 0.11	0.881 [0.766; 0.942] *	0.11
Plantar Flexion Foot In (°)	15.16 ± 4.60	15.43 ± 3.98	0.863 [0.726; 0.934] *	4.38	15.71 ± 4.88	15.37 ± 4.48	0.646 [0.376; 0.815] *	7.67
Impact Force (kN)	1.60 ± 0.31	1.63 ± 0.30	0.963 [0.909; 0.984] *	0.16	1.78 ± 0.32	1.78 ± 0.30	0.961 [0.914; 0.982] *	0.17
Leg Stiffness (kN/m)	9.87 ± 2.04	9.96 ± 1.82	0.952 [0.898; 0.978] *	1.17	10.14 ± 2.23	10.35 ± 2.34	0.717 [0.469; 0.862] *	3.38

**Table 2:** Mean, standard deviation, ICC values (with 95% CI), and minimal detectable change (MDC) of running parameters were measured using insoles for overground conditions. Significant results are indicated as follows: * = *p* < 0.001, ^+^ = *p* < 0.004.

	OVERGROUND RUNNING—INSOLES							
	Comfortable Speed				Fast Speed			
	Mean ± SD		ICC [95% CI]	MDC	Mean ± SD		ICC [95% CI]	MDC
	Session 1	Session 2			Session 1	Session 2		
Speed (m/s)	2.76 ± 0.41	2.88 ± 0.33	0.514 [0.192; 0.738] ^+^	0.73	3.98 ± 0.50	4.02 ± 0.42	0.687 [0.439; 0.838] *	0.71
Stride Cadence (step/min)	79.83 ± 3.08	80.31 ± 2.87	0.745 [0.524; 0.873] *	4.14	90.02 ± 6.74	90.27 ± 6.41	0.662 [0.399; 0.824] *	10.51
Flight Time (s)	0.21 ± 0.06	0.22 ± 0.05	0.859 [0.694; 0.935] *	0.06	0.25 ± 0.04	0.25 ± 0.04	0.928 [0.855; 0.965] *	0.03
Stance Time (s)	0.27 ± 0.03	0.27 ± 0.03	0.758 [0.512; 0.884] *	0.04	0.21 ± 0.02	0.21 ± 0.02	0.718 [0.485; 0.855] *	0.03
Stride Time (s)	0.75 ± 0.03	0.75 ± 0.03	0.745 [0.525; 0.873] *	0.04	0.67 ± 0.05	0.67 ± 0.05	0.685 [0.434; 0.837] *	0.07
Swing Time (s)	0.48 ± 0.03	0.48 ± 0.03	0.883 [0.764; 0.944] *	0.03	0.46 ± 0.04	0.46 ± 0.04	0.798 [0.617; 0.898] *	0.05
Loading Time (s)	0.12 ± 0.02	0.12 ± 0.01	0.645 [0.362; 0.820] *	0.02	0.10 ± 0.01	0.09 ± 0.01	0.677 [0.402; 0.840] *	0.02
Propulsion Time (s)	0.15 ± 0.02	0.15 ± 0.02	0.655 [0.379; 0.825] *	0.03	0.12 ± 0.01	0.12 ± 0.01	0.531 [0.190; 0.758] ^+^	0.02
Stride Length (m)	2.07 ± 0.29	2.16 ± 0.26	0.628 [0.341; 0.808] *	0.47	2.66 ± 0.25	2.67 ± 0.22	0.844 [0.700; 0.922] *	0.25
Stride Height (m)	0.27 ± 0.10	0.30 ± 0.09	0.700 [0.451; 0.848] *	0.14	0.46 ± 0.11	0.46 ± 0.12	0.887 [0.776; 0.944] *	0.11
Plantar Flexion Foot In (°)	21.59 ± 4.75	21.97 ± 4.64	0.885 [0.770; 0.945] *	4.37	21.17 ± 5.29	20.63 ± 4.87	0.612 [0.328; 0.794] *	8.72
Impact Force (kN)	1.46 ± 0.29	1.46 ± 0.26	0.935 [0.863; 0.970] *	0.27	1.69 ± 0.32	1.69 ± 0.30	0.958 [0.909; 0.981] *	0.17
Leg Stiffness (kN/m)	10.00 ± 2.15	9.74 ± 1.80	0.749 [0.524; 0.876] *	2.74	10.14 ± 1.99	10.55 ± 2.11	0.672 [0.396; 0.838] *	3.24

**Table 3:** Mean, standard deviation, ICC values (with 95% CI), and minimal detectable change (MDC) of running parameters measured using a motion-capture system for treadmill conditions. Significant results are indicated as follows: * = *p* < 0.001.

	TREADMILL RUNNING—MOTION CAPTURE							
	Comfortable Speed				Fast Speed			
	Mean ± SD		ICC [95% CI]	MDC	Mean ± SD		ICC [95% CI]	MDC
	Session 1	Session 2			Session 1	Session 2		
Speed (m/s)	2.35 ± 0.31	2.35 ± 0.32	0.999 [0.997; 0.999] *	0.03	2.88 ± 0.32	2.88 ± 0.32	0.997 [0.994; 0.999] *	0.05
Stride Cadence (step/min)	78.64 ± 4.31	79.53 ± 3.86	0.877 [0.728; 0.943] *	3.97	81.28 ± 3.97	81.40 ± 4.13	0.919 [0.837; 0.961] *	3.17
Flight Time (s)	0.14 ± 0.06	0.13 ± 0.07	0.954 [0.905; 0.978] *	0.04	0.20 ± 0.05	0.19 ± 0.06	0.924 [0.848; 0.963] *	0.04
Stance Time (s)	0.32 ± 0.04	0.31 ± 0.04	0.950 [0.899; 0.976] *	0.02	0.27 ± 0.03	0.27 ± 0.03	0.948 [0.895; 0.975] *	0.02
Stride Time (s)	0.77 ± 0.04	0.76 ± 0.04	0.884 [0.734; 0.947] *	0.04	0.74 ± 0.04	0.74 ± 0.04	0.934 [0.867; 0.968] *	0.03
Swing Time (s)	0.45 ± 0.04	0.44 ± 0.04	0.927 [0.822; 0.968] *	0.03	0.47 ± 0.04	0.47 ± 0.04	0.920 [0.840; 0.961] *	0.03
Stride Length (m)	1.69 ± 0.20	1.67 ± 0.21	0.982 [0.957; 0.992] *	0.08	1.98 ± 0.20	1.98 ± 0.21	0.985 [0.970; 0.993] *	0.07
Stride Height (m)	0.26 ± 0.06	0.26 ± 0.06	0.928 [0.855; 0.965] *	0.04	0.35 ± 0.07	0.34 ± 0.08	0.914 [0.828; 0.958] *	0.06
Plantar Flexion Foot In (°)	13.39 ± 3.39	13.28 ± 3.22	0.853 [0.715; 0.927] *	3.48	13.67 ± 4.03	14.09 ± 3.83	0.886 [0.777; 0.944] *	3.64

**Table 4:** Mean, standard deviation, ICC values (with 95% CI), and minimal detectable change (MDC) of running parameters measured using insoles for treadmill conditions. Significant results are indicated as follows: * = *p* < 0.001.

	TREADMILL RUNNING—INSOLES							
	Comfortable Speed				Fast Speed			
	Mean ± SD		ICC [95% CI]	MDC	Mean ± SD		ICC [95% CI]	MDC
	Session 1	Session 2			Session 1	Session 2		
Speed (m/s)	2.36 ± 0.30	2.35 ± 0.30	0.995 [0.989; 0.998] *	0.06	2.86 ± 0.30	2.86 ± 0.31	0.990 [0.979; 0.995] *	0.08
Stride Cadence (step/min)	78.66 ± 4.31	79.56 ± 3.85	0.875 [0.725; 0.942] *	3.99	81.26 ± 3.97	81.37 ± 4.12	0.919 [0.837; 0.961] *	3.17
Flight Time (s)	0.17 ± 0.05	0.16 ± 0.06	0.906 [0.814; 0.954] *	0.05	0.21 ± 0.05	0.21 ± 0.06	0.903 [0.807; 0.952] *	0.05
Stance Time (s)	0.30 ± 0.04	0.30 ± 0.04	0.897 [0.796; 0.949] *	0.03	0.26 ± 0.02	0.27 ± 0.03	0.931 [0.861; 0.967] *	0.02
Stride Time (s)	0.77 ± 0.04	0.76 ± 0.04	0.883 [0.736; 0.946] *	0.04	0.74 ± 0.04	0.74 ± 0.04	0.935 [0.867; 0.968] *	0.03
Swing Time (s)	0.46 ± 0.04	0.45 ± 0.04	0.881 [0.760; 0.942] *	0.04	0.48 ± 0.04	0.48 ± 0.04	0.907 [0.815; 0.955] *	0.04
Stride Length (m)	1.80 ± 0.21	1.78 ± 0.22	0.983 [0.948; 0.993] *	0.08	2.11 ± 0.21	2.11 ± 0.22	0.985 [0.969; 0.993] *	0.07
Stride Height (m)	0.23 ± 0.07	0.23 ± 0.07	0.920 [0.841; 0.961] *	0.05	0.34 ± 0.08	0.33 ± 0.09	0.910 [0.821; 0.956] *	0.07
Plantar Flexion Foot In (°)	19.53 ± 3.80	19.05 ± 3.96	0.888 [0.779; 0.945] *	3.57	19.17 ± 4.46	19.29 ± 4.47	0.922 [0.843; 0.962] *	3.42

**Table 5:** Mean, standard deviation, and ICC values (with 95% CI) of running parameters for overground conditions. Significant results are indicated as follows: * = *p* < 0.001.

	OVERGROUND RUNNING					
	Comfortable Speed			Fast Speed		
	Mean ± SD		ICC [95% CI]	Mean ± SD		ICC [95% CI]
	Motion Capture	Insoles		Motion Capture	Insoles	
Speed (m/s)	2.81 ± 0.34	2.81 ± 0.32	0.987 [0.972; 0.994] *	4.15 ± 0.55	4.00 ± 0.42	0.948 [0.894; 0.975] *
Stride Cadence (step/min)	79.96 ± 3.07	79.94 ± 2.85	0.968 [0.934; 0.985] *	87.89 ± 5.23	90.21 ± 5.99	0.908 [0.816; 0.955] *
Flight Time (s)	0.20 ± 0.06	0.21 ± 0.05	0.965 [0.928; 0.983] *	0.26 ± 0.04	0.25 ± 0.04	0.940 [0.878; 0.971] *
Stance Time (s)	0.27 ± 0.03	0.27 ± 0.03	0.971 [0.940; 0.986] *	0.21 ± 0.02	0.21 ± 0.02	0.953 [0.903; 0.977] *
Stride Time (s)	0.75 ± 0.03	0.75 ± 0.03	0.977 [0.952; 0.989] *	0.68 ± 0.04	0.67 ± 0.04	0.950 [0.898; 0.976] *
Swing Time (s)	0.48 ± 0.04	0.48 ± 0.03	0.952 [0.902; 0.977] *	0.47 ± 0.04	0.46 ± 0.04	0.918 [0.836; 0.960] *
Loading Time (s)	0.13 ± 0.01	0.12 ± 0.01	0.858 [0.723; 0.930] *	0.11 ± 0.01	0.09 ± 0.01	0.811 [0.640; 0.905] *
Propulsion Time (s)	0.15 ± 0.02	0.15 ± 0.02	0.910 [0.821; 0.956] *	0.10 ± 0.02	0.12 ± 0.01	0.760 [0.555; 0.878] *
Stride Length (m)	2.12 ± 0.27	2.11 ± 0.24	0.979 [0.956; 0.990] *	2.86 ± 0.35	2.66 ± 0.22	0.849 [0.707; 0.925] *
Stride Height (m)	0.31 ± 0.08	0.28 ± 0.09	0.974 [0.947; 0.988] *	0.49 ± 0.11	0.46 ± 0.11	0.967 [0.932; 0.984] *
Plantar Flexion Foot In (°)	15.53 ± 4.04	21.98 ± 4.36	0.922 [0.842; 0.962] *	15.47 ± 4.21	20.85 ± 4.53	0.891 [0.784; 0.947] *
Impact Force (kN)	1.62 ± 0.30	1.47 ± 0.27	0.973 [0.944; 0.987] *	1.78 ± 0.31	1.70 ± 0.30	0.957 [0.912; 0.979] *
Leg Stiffness (kN/m)	9.87 ± 1.86	9.81 ± 1.79	0.917 [0.833; 0.960] *	10.40 ± 2.28	10.47 ± 1.91	0.729 [0.504; 0.861] *

**Table 6:** Mean, standard deviation, and ICC values (with 95% CI) of running parameters for treadmill conditions. Significant results are indicated as follows: * = *p* < 0.001.

	TREADMILL RUNNING					
	Comfortable Speed			Fast Speed		
	Mean ± SD		ICC [95% CI]	Mean ± SD		ICC [95% CI]
	Motion Capture	Insoles		Motion Capture	Insoles	
Speed (m/s)	2.35 ± 0.32	2.36 ± 0.30	0.996 [0.991; 0.998] *	2.88 ± 0.32	2.86 ± 0.30	0.986 [0.971; 0.993] *
Stride Cadence (step/min)	79.10 ± 3.98	79.12 ± 3.98	0.999 [0.999; 0.999] *	81.35 ± 3.97	81.32 ± 3.96	0.999 [0.999; 0.999] *
Flight Time (s)	0.13 ± 0.07	0.16 ± 0.06	0.915 [0.830; 0.959] *	0.19 ± 0.05	0.21 ± 0.05	0.948 [0.894; 0.975] *
Stance Time (s)	0.31 ± 0.04	0.30 ± 0.04	0.948 [0.893; 0.975] *	0.27 ± 0.02	0.26 ± 0.03	0.940 [0.878; 0.971] *
Stride Time (s)	0.76 ± 0.04	0.76 ± 0.04	0.999 [0.999; 0.999] *	0.74 ± 0.04	0.74 ± 0.04	0.999 [0.999; 0.999] *
Swing Time (s)	0.45 ± 0.04	0.46 ± 0.04	0.962 [0.923; 0.982] *	0.47 ± 0.04	0.48 ± 0.04	0.977 [0.952; 0.989] *
Stride Length (m)	1.68 ± 0.20	1.79 ± 0.22	0.994 [0.987; 0.997] *	1.98 ± 0.21	2.11 ± 0.22	0.990 [0.979; 0.995] *
Stride Height (m)	0.26 ± 0.06	0.23 ± 0.06	0.981 [0.961; 0.991] *	0.35 ± 0.07	0.33 ± 0.08	0.981 [0.961; 0.991] *
Plantar Flexion Foot In (°)	13.33 ± 3.18	19.29 ± 3.77	0.899 [0.799; 0.951] *	13.88 ± 3.82	19.23 ± 4.37	0.919 [0.836; 0.960] *

## Data Availability

Data are available on request due to restrictions (e.g., privacy and ethics).

## Supplementary Materials

The following supporting information can be downloaded at: https://www.mdpi.com/article/10.3390/s24165435/s1, Table S1: Calculation of running parameters for motion-capture system; Table S2: Comparison of absolute and relative error between the two sessions of each parameter extracted with motion capture and with insoles for overground running; Table S3: Comparison of absolute and relative error between the two sessions of each parameter extracted with motion capture and with insoles for treadmill running; Table S4: Comparison between intraclass and concordance correlation coefficient for overground and treadmill running; Figure S1: Lin’s concordance correlation plot for all running parameters during fast and comfortable overground running; Figure S2: Lin’s concordance correlation plot for all running parameters during fast and comfortable treadmill running. References [38,39,40,41,42] are cited in Supplementary Materials.
