# Supplementary material for: Validity and Test–Retest Reliability of Spatiotemporal Running Parameter Measurement Using Embedded Inertial Measurement Unit Insoles
Source: Sensors (Basel). 2024 Aug 22;24(16):5435. doi: 10.3390/s24165435 (PMC11359420; doi:10.3390/s24165435)

Supplementary Data

Table S1. Calculation of running parameters for motion capture system.

Parameters	Calculation and extraction in motion capture
Speed (m/s)	Walkway: Antero-posterior speed of the estimated center of mass (calculated with the resultant of the four pelvis markers: RASI, LASI, RPSI, and LPSI).<br>Treadmill: Absolute average of the minimal antero-posterior velocity of each heel marker (R/LHEE) plus the root mean square (RMS) of the velocity of each heel marker [38].
Stride Cadence (step/min)	Step frequency per minute (60 / step time / 2).
Flight Time (s)	Sum of time between Toe Off and contralateral Heel Strike, and time between contralateral Toe Off and final Heel Strike.
Stance Time (s)	Time between initial Heel Strike and Toe Off of the same side.
Stride Time (s)	Time between initial Heel Strike and final Heel Strike of the same side.
Swing Time (s)	Time between Toe Off and final Heel Strike of the same side.
Loading Time (s)	Time between Heel Strike and Mid Stance of the same side [39].
Propulsion Time (s)	Time between Mid Stance and Heel Strike of the same side.
Stride Length (m)	Walkway: Resultant of the antero-posterior and mediolateral distances between the heel marker at the initial Heel Strike and the heel marker at the following Heel Strike.<br>Treadmill: Sum of the first step length (antero-posterior distance between the two heel markers at time of contralateral Heel Strike) and the second step length (antero-posterior distance between the two heel markers at time of the final Heel Strike) [40]. For each step, we added to the step length the push-off length, which is an estimation of the antero-posterior distance traveled by the heel marker (R/LHEE) if the foot was flat on the ground before the rotation of the ankle [41].
Stride Height (m)	*Walkway:* Maximum vertical value of a virtual marker close to the arch during swing phase.<br>*Treadmill:* Difference between maximum and minimum of vertical value of a virtual marker close to the arch during swing phase plus mean vertical value of a virtual marker close to the arch during static calibration.
Plantar Flexion Foot In (°)	Angle between the foot vector (projected into the laboratory's transverse plane) and the transverse laboratory axis at the time of initial Heel Strike.
Impact Force (kN)	Maximum vertical ground reaction force during the stance phase.
Leg Stiffness (kN/m)	Ratio of the impact force to the vertical displacement of the center of mass [42].

Table S2. Comparison of absolute and relative error between the two sessions of each parameter extracted with motion capture and with insoles for overground running. * = $p < 0.004$.

	OVERGROUND RUNNING												
	Comfortable speed						Fast speed						
	Absolute error			Relative error (%)			Absolute error			Relative error (%)			
	Motion Capture	Insoles		Motion Capture	Insoles		Motion Capture	Insoles		Motion Capture	Insoles		
	**Mean ± SD**	**Mean ± SD**	***p***	**Mean ± SD**	**Mean ± SD**	***p***	**Mean ± SD**	**Mean ± SD**	**p**	**Mean ± SD**	**Mean ± SD**	**p**	
Speed (m.s^{-1})	0.28 ± 0.26	0.27 ± 0.26	0.630	9.2 ± 7.9	9.0 ± 7.8	0.468	0.29 ± 0.26	0.29 ± 0.23	0.947	6.7 ± 5.9	5.4 ± 7.1	0.543	
Stride Cadence (step.min^{-1})	1.23 ± 1.18	1.64 ± 1.39	0.016	1.5 ± 1.5	2.1 ± 1.8	0.018	2.89 ± 3.12	3.88 ± 3.79	0.004	3.2 ± 3.4	4.0 ± 4.2	0.006	
Flight Time (s)	0.03 ± 0.02	0.02 ± 0.02	0.062	12.5 ± 10.8	9.5 ± 9.8	0.015	0.01 ± 0.01	0.01 ± 0.01	0.386	5.5 ± 4.2	3.8 ± 4.9	0.458	
Stance Time (s)	0.02 ± 0.01	0.02 ± 0.01	0.156	6.8 ± 5.7	6.1 ± 5.1	0.202	0.01 ± 0.01	0.01 ± 0.01	0.931	6.5 ± 5.2	4.4 ± 6.4	0.887	
Stride Time (s)	0.01 ± 0.01	0.02 ± 0.01	0.011	1.6 ± 1.5	2.0 ± 1.7	0.014	0.02 ± 0.02	0.03 ± 0.03	0.009	3.5 ± 3.7	4.1 ± 4.3	0.006	
Swing Time (s)	0.01 ± 0.01	0.01 ± 0.01	0.977	2.6 ± 2.2	2.5 ± 1.9	0.909	0.02 ± 0.01	0.02 ± 0.02	0.271	3.5 ± 2.9	3.7 ± 4.1	0.158	
Loading Time (s)	0.01 ± 0.01	0.01 ± 0.01	0.007	5.5 ± 4.3	8.7 ± 5.9	0.005	0.01 ± 0.01	0.01 ± 0.01	0.443	7.9 ± 8.7	5.8 ± 7.1	0.681	
Propulsion Time (s)	0.02 ± 0.01	0.01 ± 0.01	0.008	12.0 ± 9.9	7.9 ± 6.1	0.007	0.01 ± 0.01	0.01 ± 0.01	0.051	13.5 ± 14.2	5.1 ± 7.8	0.026	
Stride Length (m)	0.19 ± 0.19	0.18 ± 0.17	0.219	8.2 ± 7.4	7.8 ± 6.8	0.226	0.13 ± 0.09	0.10 ± 0.08	0.059	4.6 ± 3.0	3.0 ± 3.9	0.163	
Stride Height (m)	0.05 ± 0.05	0.05 ± 0.05	0.053	12.7 ± 10.2	16.3 ± 12.5	0.006	0.04 ± 0.04	0.04 ± 0.03	0.663	8.3 ± 7.0	6.6 ± 9.1	0.304	
Plantar Flexion Foot In (°)	1.69 ± 1.52	1.51 ± 1.69	0.406	12.8 ± 15.2	8.4 ± 12.2	0.009	2.71 ± 2.89	2.92 ± 3.44	0.440	21.3 ± 29	23.8 ± 16.3	0.028	
Impact Force (N)	65.15 ± 52.88	83.49 ± 58.58	0.056	4.2 ± 3.3	5.6 ± 3.8	0.041	68.03 ± 55.08	69.78 ± 59.69	0.863	3.8 ± 2.8	3.0 ± 4.0	0.676	
Leg Stiffness (N/m)	477.43 ± 378.81	1046.77 ± 981.13	0.005	4.9 ± 3.9	11.0 ± 10.1	0.006	1250.92 ± 1099.2	1328.23 ± 979.86	0.779	11.3 ± 8.7	7.8 ± 12.2	0.661	

Table S3. Comparison of absolute and relative error between the two sessions of each parameter extracted with motion capture and with insoles for treadmill running. * = p < 0.004.

	TREADMILL RUNNING											
	**Comfortable speed**						**Fast speed**					
	**Absolute error**			**Relative error (%)**			**Absolute error**			**Relative error (%)**		
	**Motion Capture**	**Insoles**		**Motion Capture**	**Insoles**		**Motion Capture**	**Insoles**		**Motion Capture**	**Insoles**	
	**Mean ± SD**	**Mean ± SD**	***p***	**Mean ± SD**	**Mean ± SD**	***p***	**Mean ± SD**	**Mean ± SD**	**p**	**Mean ± SD**	**Mean ± SD**	**p**
Speed ($m.s^{-1}$)	0.01 ± 0.01	0.02 ± 0.02	0.162	0.5 ± 0.4	0.7 ± 0.9	0.198	0.02 ± 0.01	0.03 ± 0.03	0.054	0.6 ± 0.5	1.0 ± 1.1	0.060
Stride Cadence ($step.min^{-1}$)	1.61 ± 1.30	1.60 ± 1.32	0.780	2.0 ± 1.6	2.0 ± 1.6	0.753	1.30 ± 1.00	1.31 ± 0.99	0.847	1.6 ± 1.2	1.2 ± 1.6	0.807
Flight Time (s)	0.02 ± 0.01	0.02 ± 0.02	0.515	22.4 ± 31.5	13.3 ± 13.4	0.052	0.02 ± 0.01	0.02 ± 0.01	0.114	9.5 ± 8.3	8.6 ± 10.0	0.559
Stance Time (s)	0.01 ± 0.01	0.01 ± 0.01	0.008	2.4 ± 2.6	3.6 ± 3.6	0.007	0.01 ± 0.01	0.01 ± 0.01	0.060	2.1 ± 1.9	2.3 ± 2.8	0.008
Stride Time (s)	0.02 ± 0.01	0.02 ± 0.01	0.173	2.1 ± 1.7	2.1 ± 1.7	0.175	0.01 ± 0.01	0.01 ± 0.01	0.917	1.6 ± 1.2	1.2 ± 1.6	0.858
Swing Time (s)	0.01 ± 0.01	0.02 ± 0.01	0.049	3.0 ± 2.3	3.8 ± 3.0	0.053	0.01 ± 0.01	0.01 ± 0.01	0.255	2.8 ± 2.1	2.4 ± 3.0	0.374
Stride Length (m)	0.03 ± 0.03	0.03 ± 0.03	0.191	1.8 ± 1.7	1.8 ± 1.6	0.967	0.03 ± 0.02	0.03 ± 0.02	0.644	1.4 ± 1.1	1.1 ± 1.4	0.936
Stride Height (m)	0.02 ± 0.02	0.02 ± 0.02	0.087	5.7 ± 5.4	7.1 ± 6.8	0.010	0.02 ± 0.02	0.03 ± 0.03	0.006	6.5 ± 7.3	9.5 ± 8.2	0.002*
Plantar Flexion Foot In (°)	1.49 ± 1.00	1.45 ± 1.15	0.854	12.1 ± 9	9.3 ± 11.4	0.191	1.54 ± 1.09	1.44 ± 1.03	0.648	12.1 ± 9.7	7.1 ± 8.4	0.030

Table S4. Comparison between intraclass (ICC) and concordance (CCC) correlation coefficient for overground and treadmill running.

	**OVERGROUND RUNNING**				**TREADMILL RUNNING**			
	**Comfortable speed**		**Fast speed**		**Comfortable speed**		**Fast speed**	
	**CCC**	**ICC**	**CCC**	**ICC**	**CCC**	**ICC**	**CCC**	**ICC**
Speed	0.986	0.987	0.903	0.948	0.996	0.996	0.984	0.986
Stride Cadence	0.968	0.968	0.834	0.908	0.999	0.999	0.999	0.999
Flight Time	0.956	0.965	0.918	0.940	0.816	0.915	0.899	0.948
Stance Time	0.965	0.971	0.950	0.953	0.902	0.948	0.885	0.940
Stride Time	0.977	0.977	0.903	0.950	0.999	0.999	0.999	0.999
Swing Time	0.949	0.952	0.842	0.918	0.929	0.962	0.953	0.977
Loading Time	0.832	0.858	0.467	0.811	-	-	-	-
Propulsion Time	0.910	0.910	0.453	0.760	-	-	-	-
Stride Length	0.978	0.979	0.690	0.849	0.871	0.994	0.825	0.990
Stride Height	0.906	0.974	0.922	0.967	0.895	0.981	0.968	0.981
Plantar Flexion Foot In	0.416	0.922	0.500	0.891	0.358	0.899	0.489	0.919
Impact Force	0.840	0.973	0.918	0.957	-	-	-	-
Leg Stiffness	0.916	0.917	0.728	0.729	-	-	-	-

Figure S1. Lin's concordance correlation plot for all running parameters during fast (black points) and comfortable (red points) overground running. Solid line = identity function.

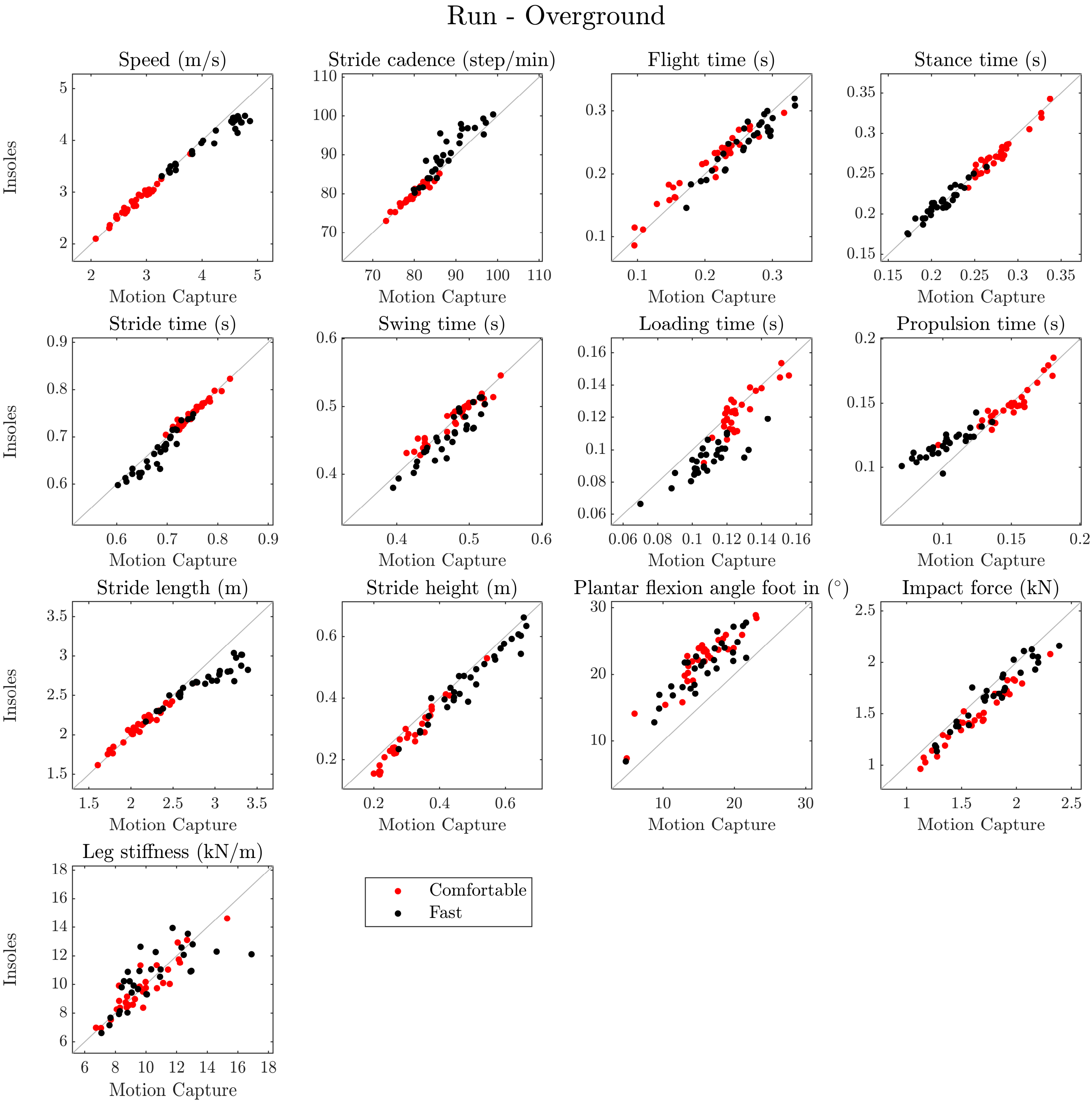

Figure S2. Lin's concordance correlation plot for all running parameters during fast (black points) and comfortable (red points) treadmill running. Solid line = identity function.

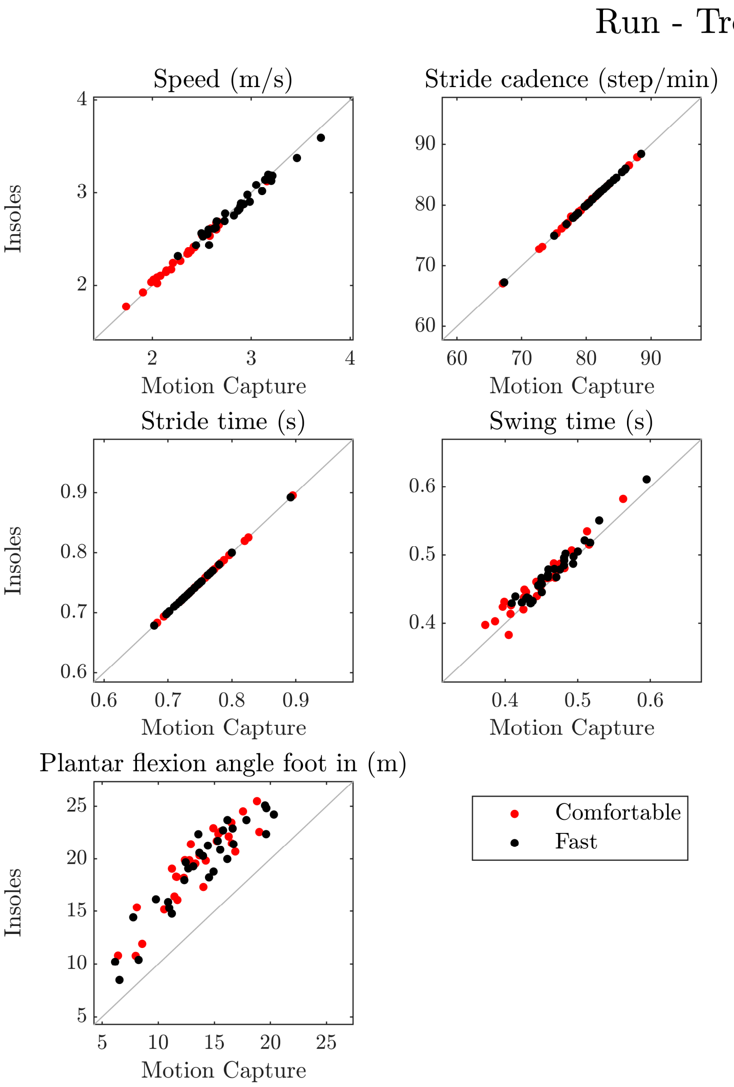

Supplement: Supplementary file 1 [file sensors-24-05435-s001.zip › sensors-3072856-supplementary.pdf]
